# A 72-Year-Old Patient with Longstanding, Untreated Familial Hypercholesterolemia but no Coronary Artery Calcification: A Case Report

**DOI:** 10.7759/cureus.2452

**Published:** 2018-04-09

**Authors:** Kipp W Johnson, Joel T Dudley, Jason R Bobe

**Affiliations:** 1 Institute for Next Generation Healthcare, Icahn School of Medicine at Mount Sinai, New York, N.Y., USA; 2 Institute for Next Generation Healthcare, Icahn School of Medicine at Mount Sinai, New York, USA; 3 Icahn Institute, Genetic and Genomic Sciences, Icahn School of Medicine at Mount Sinai, New York, USA

**Keywords:** incomplete penetrance, coronary artery calcification, familial hypercholesterolemia, cardiovascular disease, ldlr mutation, genetic resilience, high cholesterol, positive outlier, resilience

## Abstract

Familial hypercholesterolemia (FH) is a genetic disease associated with persistently elevated levels of low-density lipoprotein cholesterol (LDL-C), which ultimately leads to greatly increased rates of atherosclerosis and cardiovascular disease. Atherosclerosis progression can be clinically approximated through measurement of coronary artery calcification (CAC). CAC can be measured via electron beam computed tomography (EBCT), multi-slice computed tomography (MSCT), or contrast-enhanced CT coronary angiography (CTCA). Here, we present the case of a 72-year-old man with known FH and established hypercholesterolemia who has consistently tested negative for any significant CAC.

## Introduction

Classical familial hypercholesterolemia (FH) is an autosomal semidominant genetic disease characterized primarily by persistent elevations (>310 mg/dL) in low-density lipoprotein cholesterol (LDL-C) [1]. FH is most often caused by mutations in the gene encoding the LDL receptor(*LDLR*) and phenotypic penetrance for damaging variants is nearly complete, with approximately 90% of heterozygotes eventually developing FH [2]. Elevated levels of LDL-C have been shown via epidemiological and genetic studies to cause atherosclerosis and coronary artery disease (CAD). Consequently, both treated and untreated familial hypercholesterolemia are associated with greatly elevated risk for CAD, with estimated odds ratios of 10.3 (95 CI: 7.8-13.8) and 13.2 (10.0-17.4) respectively compared to non-FH patients [3]. Most patients with monogenic FH are heterozygous, and individuals with homozygous mutations display an even more severe damaging phenotype [4]. More recently, it has been suggested that a significant fraction of individuals with FH and no apparent monogenic mutation may have a polygenic etiology resulting from an excess of mildly deleterious variants in a number of genes, although the true proportion of FH with this polygenic etiology is still an area of active research.

Coronary artery calcification (CAC) is an early sign of the presence of subclinical atherosclerotic disease. CAC, typically assessed via cardiac computed tomography (CT) or electron beam CT, is often observed early in patients with FH due to increased LDL-C burden [5]. Here, we present the case of a 72-year-old patient with untreated FH but no evidence of CAD on six separate CAC tests between late 1990s and 2018.

## Case presentation

Our patient is a normotensive (clinic blood pressure: 110/70) 72-year-old man with a body mass index (BMI) of 25.0 who is heterozygous for a known pathogenic mutation in *LDLR* (rs879254637) originally reported in 2007 [6]. This mutation consists of a 3-basepair deletion and 14-basepair insertion at position 11,105,586 (GRCh38.p7 reference genome) resulting in a frameshift mutation with a new stop codon at position 42 and ultimately a nonfunctional *LDLR* protein product. This is a rare variant, as there are no other examples of this variant in the gnomAD genome database [7].

The subject has a longstanding history of hypercholesterolemia. He was initially diagnosed while in his first or second year as a college student after presenting with corneal arcus and LDL-C levels above 300 mg/dL. His initial therapy consisted of clofibrate. He later switched to six packets per day of cholestyramine and niacin (nicotinic acid) which he tolerated poorly and which were ultimately discontinued when he switched to statins. In the 1980s, he participated in early trials for statins (3-hydroxy-3-methylglutaryl-CoA (HMG-CoA) reductase inhibitors) in the United States and from the 1980s through 2006 he was treated with a statin therapy of progressively increasing dosage and intensity. When atorvastatin became commercially available he switched to it at maximum dosage (80 mg), in combination with niacin for a short while. He reports that pharmacologic therapy with statins was largely ineffective at reducing his LDL-C levels, with the majority of lab results reporting results above 300 mg/dL and a single lowest value of 260 mg/dL while on combination atorvastatin and niacin. In addition to FH-directed therapy, our subject reports occasionally using baby aspirin (81 mg) and over-the-counter Vitamin D supplements and multivitamins.

**Figure 1:**
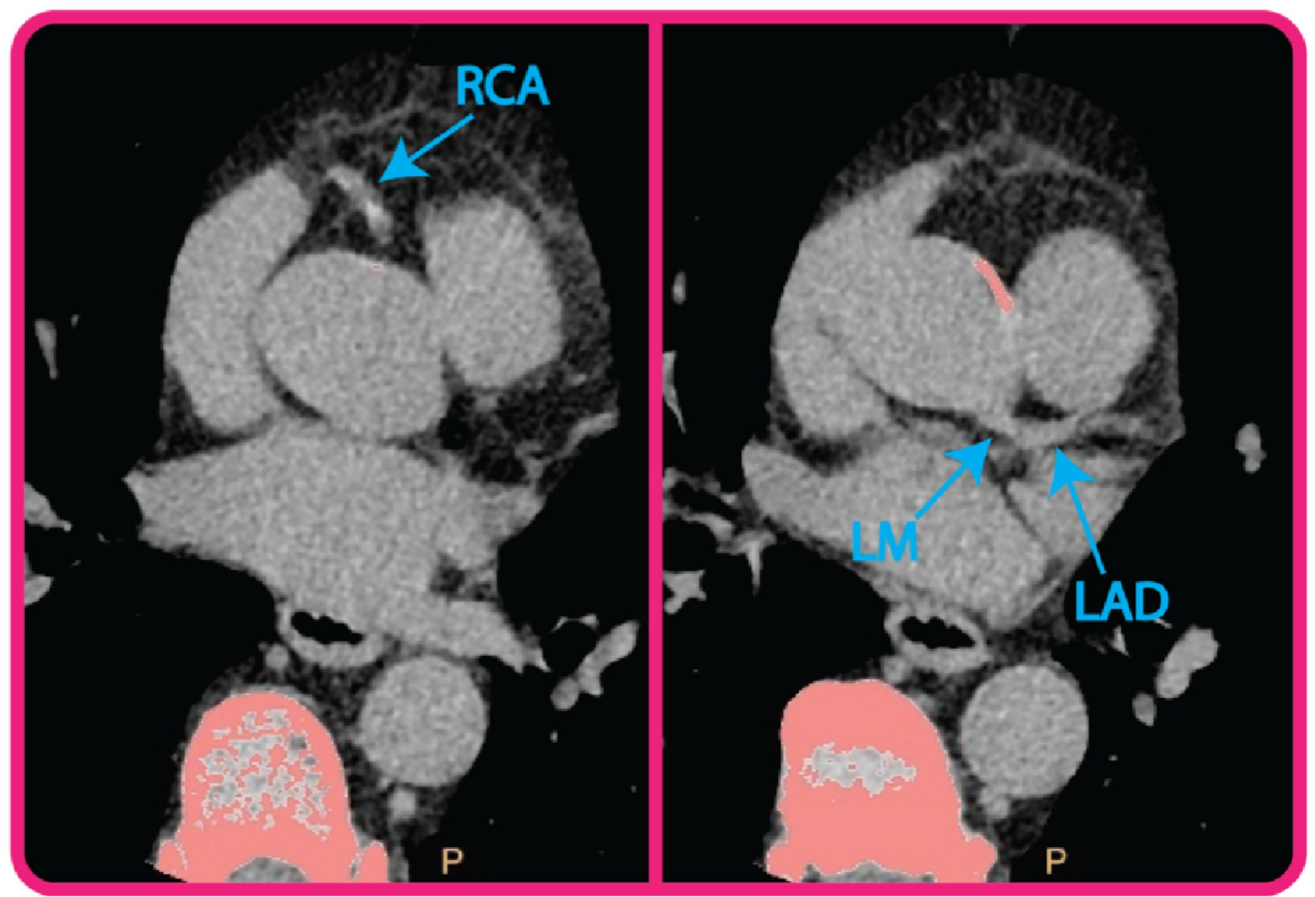
Cardiac computed tomography (CT) results. Pictured are two CT transverse slices from 2018 (patient age: 72) demonstrating the absence of calcification in the right coronary artery (RCA) and left main (LM) and left anterior descending (LAD) coronary arteries.

In the early 1990s, our patient underwent electron beam computed tomography (EBCT) imaging for CAC following a series of elevated lipid panels. Presence of CAC was assessed in the left main, left anterior descending, left circumflex, and right coronary arteries and scored using the Agatston score. His initial score was 0.0, implying a greater than 95% chance of absence of coronary artery disease. Because of this surprising finding, he subsequently undertook four additional EBCT tests from 2006 to 2014 resulting in Agatston scores of 1.6, 2.1, 0.0, and 0.0, suggesting a nearly complete absence of any coronary artery calcification. In February of 2018, he underwent multi-slice CT which revealed a complete absence of coronary artery calcification, although mild aortic calcification was observed (Figure 1). His Agatston score was again 0.

We obtained detailed lipid testing results taken between 2009 and 2018 (Table 1). In his panels between 2009 and 2018, LDL-C concentrations were measured at 486.9 ± 30 mg/dL. Our patient also underwent advanced lipid panel testing (which revealed elevated levels of apolipoprotein B (ApoB, 297.8 ± 17.6 mg/dL), mildly elevated levels of lipoprotein-associated phospholipase A2 (Lp-PLA2, 212.8 ± 4.6 ng/mL), and mildly elevated triglycerides (134.6 ± 38.9 mg/dL). The patient’s mean high-density lipoprotein (HDL) was 67.5 ± 7.2 mg/dL. Mean lipoprotein(a) (Lp(a)) levels were 4 ± 1.4 mg/dL. LDL particle subtyping revealed normal values for atherogenic LDL particles IIIa and IVb, and the majority of LDL was of the large Type I LDL. Complete blood count and comprehensive metabolic panel were generally normal except for mildly elevated blood urea nitrogen (25 mg/dL). Genotyping at *ApoE* (3/3) and *KIF6* (Trp/Trp) revealed the absence of risk alleles. No pathogenic variants were identified in the genes* PCSK9*, *APOB*, or *LDLRAP1*.

**Table 1:** Documented lab values with corresponding dates. LDL: Low-density lipoprotein; HDL: High-density lipoprotein; LP(a): Lipoprotein (a); LP-PLA2: Lipoprotein-associated phospholipase A2; ApoB: Apolipoprotein B; Q-LDL: Quantitative LDL.

(A) Longitudinal Laboratory Values								
Date	2009	2010	2011	2012	2013	2014	2018	Mean (SD)
Total Cholesterol (mg/dL)	542	578	562, 636	599	617	561	564	582.4 (31.2)
LDL (mg/dL)	450	484	470, 535	506	519	461	470	486.9 (30.0)
HDL (mg/dL)	60	69	73, 75	76	68	62	57	67.5 (7.2)
Triglycerides (mg/dL)	113	124	96, 130	86	150	191	187	132.6 (38.9)
Lp(a) (mg/dL)	6	3	-	3	4	-	-	4 (1.4)
Lp-PLA2 (ng/mL)	-	213	208	211	219	-	-	212.8 (4.6)
ApoB (mg/dL)	-	288	324	288	291	-	-	297.8 (17.6)
Insulin (µIU/mL)	-	8	5	5	8	-	17	8.6 (4.9)
LDL Pattern	A	A	A	A	A	-	-	-
LDL Particle No.	-	-	-	-	-	>3500	>3500	-
Small LDL Particle No.	-	-	-	-	-	2300	2215	2257.5 (60.1)
LDL Particle Size (Å)	-	273	278	281	276	268	214	265 (25.4)
HDL Particles, Total (µmol/L)	-	-	-	-	-	26.8	25.3	26.1 (1.1)
(B) Advanced Lipid Testing								
Date	2009	2010	2011	2012	2013	2014	2018	Mean (SD)
LDL-1 (%)	-	1.6	3.9	3.9	2.3	-	-	2.9 (1.2)
LDL-IIa (%)	-	0.1	0.5	0.3	0.4	-	-	0.3 (0.2)
LDL-IIb (%)	-	4.8	8.8	7.2	6.7	-	-	6.9 (1.6)
LDL-IIIa (%)	-	1.2	3.0	3.1	1.8	-	-	2.3 (0.9)
LDL-IIIb (%)	-	0.4	0.9	0.8	0.5	-	-	0.6 (0.2)
LDL IIIa+IIIb (%)	-	1.6	3.9	3.9	2.3	-	-	2.9 (1.2)
LDL-IVa (%)	-	0.2	0.8	0.7	0.5	-	-	0.6 (0.3)
LDL-IVb (%)	-	0.1	0.5	0.3	0.4	-	-	0.3 (0.2)
Q-LDL IIIa+IIIb (mg/dL)	-	5.6	15.0	13.4	8.0	-	-	10.5 (4.4)
Q-LDL IVb (mg/dL)	-	0.1	2.3	1.3	1.7	-	-	1.3 (0.9)
HDL-2a (%)	-	23	26	21	24	-	-	23.5 (2.1)
HDL-2b (%)	-	23	23	24	23	-	-	23.3 (0.5)
HDL-3a (%)	-	31	27	27	28	-	-	28.2 (1.9)
HDL-3b (%)	-	19	16	17	18	-	-	17.5 (1.3)
HDL-3c (%)	-	3	8	10	8	-	-	7.2 (3.0)

Of note, our subject reports that since 2006 he has eaten a high-fat, low-carbohydrate-based diet. Before this, he reports that he generally ate a low-fat, high-carbohydrate diet. He reports drinking on average three glasses of wine per week. He has never smoked and drinks five to six cups of coffee per day on average. Past medical history includes obstructive sleep apnea managed with a continuous positive airway pressure (CPAP) machine, appendicitis at age 18, diverticulitis, a ruptured Achilles tendon attributed to diverticulitis treatment with ciprofloxacin, and intestinal adhesions and surgically repaired intestinal volvulus. The patient also reports that he had a prostatectomy in 2016 related to cancer. He consistently has had blood glucose levels between 100 and 105 mg/dL for the past decade. The patient denies any significant experiences of chest pain or other symptoms implying angina or ischemia, although he has had a few isolated episodes of palpitations. He has one sister three years his senior who also has FH and a history of high lipid levels. She also has no history of myocardial infarction, angina, or other symptoms of coronary artery disease. His mother had FH, although she died of pancreatic cancer at age 77. She and her three siblings were never treated for, and had no history of, cardiovascular disease. The patient reports that his father had one high cholesterol score (290s), but was never diagnosed with FH, had no history of cardiovascular disease and died in his 80s during surgery for hernia repair. Our patient has two children, neither of whom have FH. 

## Discussion

Our patient is a 72-year-old man affected with a known heterozygous, damaging *LDLR* mutation causing familial hypercholesterolemia and the resulting expected elevated levels of serum cholesterol. Despite his lipid history, our patient has evidence for the absence of any significant atherosclerotic disease as assessed via repeated EBCT coronary artery calcification imaging over several years with Agatston scores of 0, 1, 2, 0, and 0. In comparison, it has been estimated that the median Agatston score for white males between the age of 65 and 74 is 145 [8]. In fact, our patient’s mean Agatston score of <1 is below the median for even low risk, healthy controls in the closest comparable age reference bracket for which there were large enough numbers (55-64 years old; median score 13) [8].

This is a surprising result. Patients with damaging FH-related mutations and longstanding elevations in lipid levels are expected to experience both premature CAC as well as a greater extent of CAC compared to age-matched controls [5]. CAC risk is even higher in patients with monogenic FH, as our patient possesses, when compared to patients with polygenic CAC [9]. Additionally, it has been noted that patients with a long history of high-intensity statin therapy, as with our patient, are expected to have higher levels of CAC as assessed by EBCT [10]. Our patient’s mildly elevated levels of Lp-PLA2 and ApoB are also associated with elevated CAC, although evidence for these newer biomarkers is still emerging.

Essentially, our patient demonstrates an absence of atherosclerotic disease compared to even healthy individuals despite a longstanding history of familial hypercholesterolemia and the presence of a number of cardiovascular risk factors. However, we also note that our patient demonstrates a number of protective factors and behaviors. For example, our subject has a normal BMI; is not hypertensive; has never smoked cigarettes; eats a high-fat, low-carbohydrate diet; and also possesses a strongly positive life outlook. Taken altogether, we hypothesize that a multitude of protective factors (or on the contrary, the absence of damaging risk factors) could contribute to what we have christened a “polyprotective” phenotype. Furthermore, the absence of significant cardiovascular events in his family members who also have FH implies that there could be a genetic basis for his evasion of adverse cardiovascular outcomes.

We are currently in the process of designing further assays to attempt to identify the factors protecting him from coronary artery calcification. Hypotheses involve each step in the biomolecular pathogenic process for coronary artery disease: LDL particle morphology and function, LDL oxidation and macrophage function, chronic inflammation, and vascular hemodynamics. Most tantalizingly, we intend to perform whole-genome sequencing on our subject with the hope of identifying novel mutations which could be counteracting his damaging loss-of-function mutation in *LDLR*. This would be analogous to the early 2000s discovery of Sharlayne Tracy’s mutation in the protein Proprotein convertase subtilisin/kexin type 9 (*PCSK9*), which led to the successful clinical development of the anti-lipid PCSK9-inhibiting drugs alirocumab and evolocumab.

## Conclusions

We have identified a man with a longstanding history of elevated lipid levels caused by a damaging variant in the low-density lipoprotein receptor gene. Despite his condition, our patient has a surprising complete absence of significant coronary artery calcification. Further efforts are underway to interrogate why our patient has escaped the damaging consequences of familial hypercholesterolemia and could inform future efforts in drug discovery and therapy development.
